# The phylum Vertebrata: a case for zoological recognition

**DOI:** 10.1186/s40851-018-0114-y

**Published:** 2018-12-26

**Authors:** Naoki Irie, Noriyuki Satoh, Shigeru Kuratani

**Affiliations:** 1 Department of Biological Sciences, School of Science, University of Tokyo, Tokyo, 113-0033 Japan; 2 Universal Biology Institute, University of Tokyo, Tokyo, 113-0033 Japan; 3 Marine Genomics Unit, Okinawa Institute of Science and Technology Graduate University, Onna, Okinawa, 904-0495 Japan; 4 Laboratory for Evolutionary Morphology, RIKEN Center for Biosystems Dynamics Research, and Evolutionary Morphology Laboratory, RIKEN Cluster for Pioneering Research, 2-2-3 Minatojima-minami, Chuo-ku, Kobe, 650-0047 Japan

**Keywords:** Gene family, Gene expression profile, Molecular phylogeny, Organ development, Phylum Vertebrata, Zoological classification

## Abstract

The group Vertebrata is currently placed as a subphylum in the phylum Chordata, together with two other subphyla, Cephalochordata (lancelets) and Urochordata (ascidians). The past three decades, have seen extraordinary advances in zoological taxonomy and the time is now ripe for reassessing whether the subphylum position is truly appropriate for vertebrates, particularly in light of recent advances in molecular phylogeny, comparative genomics, and evolutionary developmental biology. Four lines of current research are discussed here. First, molecular phylogeny has demonstrated that Deuterostomia comprises Ambulacraria (Echinodermata and Hemichordata) and Chordata (Cephalochordata, Urochordata, and Vertebrata), each clade being recognized as a mutually comparable phylum. Second, comparative genomic studies show that vertebrates alone have experienced two rounds of whole-genome duplication, which makes the composition of their gene family unique. Third, comparative gene-expression profiling of vertebrate embryos favors an hourglass pattern of development, the most conserved stage of which is recognized as a phylotypic period characterized by the establishment of a body plan definitively associated with a phylum. This mid-embryonic conservation is supported robustly in vertebrates, but only weakly in chordates. Fourth, certain complex patterns of body plan formation (especially of the head, pharynx, and somites) are recognized throughout the vertebrates, but not in any other animal groups. For these reasons, we suggest that it is more appropriate to recognize vertebrates as an independent phylum, not as a subphylum of the phylum Chordata.

## Background

The origin and evolution of vertebrates has long been a focus of zoological study [1]. Vertebrates were distinguished from invertebrates as early as a few hundred years BC [2]. The present zoological taxonomy classifies Vertebrata as a subphylum of the phylum Chordata, together with two other invertebrate subphyla, Cephalochordata (lancelets) and Urochordata (ascidians). The aim of this review is to discuss whether the subphylum Vertebrata is supported by data obtained from recent zoological research.

**Fig. 1:**
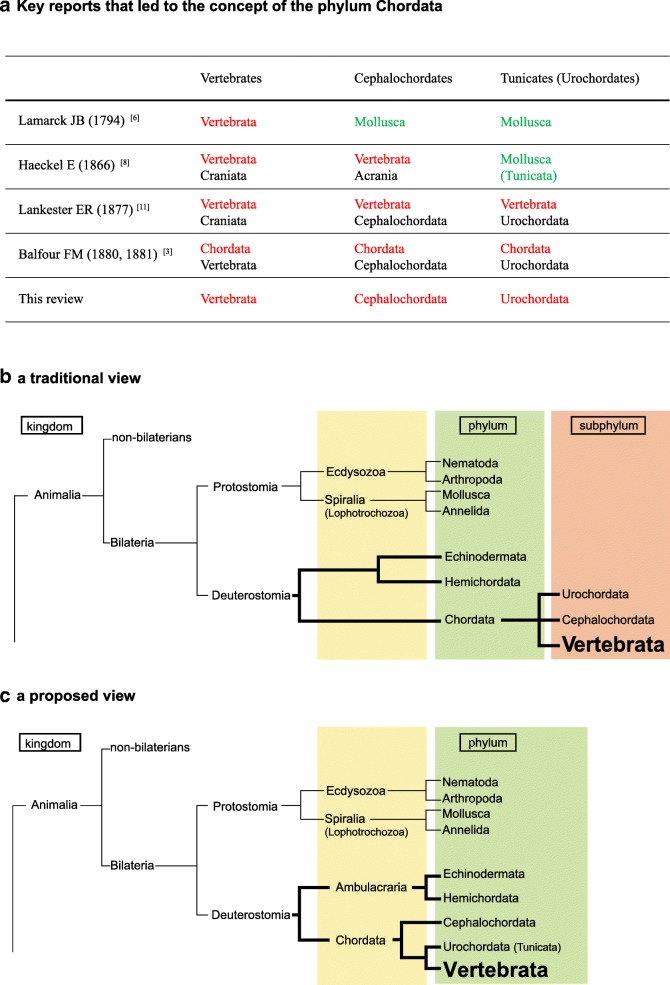
Subphylum Vertebrata of the phylum Chordata. **a** Key reports that led to the concept of the phylum Chordata. Terms in red are of phylum rank and those in black are of subphylum rank. Those in green were recognized as invertebrates at the times indicated in the first column. **b** Traditional view (upper) and **c** our proposed view (lower) of chordate phylogeny with respect to inter-phylum relationships. The proposed phylogeny regards the Cephalochordata, the Urochordata, and the Vertebrata as separate phyla, rather than as subphyla. (m*odified from* [17])

The present classifications of vertebrates was established by Balfour [3] in 1880–1881(Fig. 1a), and the subphylum rank of Vertebrata has not been the subject of critical discussion since that time. Prior to Balfour’s classification, in the mid-to-late eighteenth century, lancelets [4] and tunicates [5] were considered invertebrates and grouped with Mollusca, although Yarrell [4] noted that lancelets possess a primitive axial rod and thus show some affinity to vertebrates. In 1794, Lamarck [6] proposed the phylum Vertebrata, distinguishing them from invertebrates (Fig. 1a). The publication of Charles Darwin’s book *On the origin of species* in 1859 [7] led to vigorous discussion of animal evolution, including the classification of vertebrates. In 1866, Haeckel [8], himself a committed Darwinian, proposed a new concept for phylum Vertebrata, as comprising two subphyletic groups: vertebrates as Craniata (animals with heads) and lancelets as Acrania (animals without heads) (Fig. 1a).

In1886 and 1887, Kowalevsky reported his discovery of the notochord in ascidian larvae [9] and in lancelet adults [10]. His reports impressed zoologists with the affinity of these two invertebrates with vertebrates, as all three groups have a notochord. Following further discussion, in 1877, Lankester [11] proposed that the phylum Vertebrata consisted of three subphyla: Craniata, Cephalochordata (animals with a notochord that runs through the entire body to the tip of trunk), and Urochordata (or Tunicata, animals with a notochord that is present only in the tail) (Fig. 1a). Thus, the basic schema for the taxonomic classification of vertebrates and other notochordal taxa was fixed under Lankester’s proposed system. The following year, Balfour [3] altered the terminology of Vertebrata to Chordata and Craniata to Vertebrata (Fig. 1a), further emphasizing the notochord (and the dorsal nerve cord or neural tube); this led to the current concept of the subphylum Vertebrata in the phylum Chordata.

Over the past three decades, extraordinary advances have been made in zoological classification thanks to the incorporation of new methods and technologies, including evolutionary developmental biology (evo-devo), molecular phylogeny, and comparative genomics. Our understanding of the phylogenic position of metazoan taxa or the evolutionary relationships among bilaterian groups is now changing as a result of data obtained using these new tools. For example, protostomes are now subdivided into two major groups—lophotrochozoans (spiralians) and ecdysozoans—on the basis of their molecular phylogeny (Fig. 1b) [12, 13]. Nevertheless, the classification of the phylum Chordata and its three sub-phylum system has largely remained unchallenged, although recently a few researchers have come to question this taxonomy. For example, Swalla et al. [14] and Zeng and Swalla [15], on the basis of molecular phylogeny as determined by 18S rDNA sequence comparison, suggested that tunicates are monophyletic and should therefore be recognized as a phylum. Satoh et al. [16] proposed a three-phylum system of chordates instead of the three-subphylum system. Although this notion was viewed with interest by many zoologists, and a growing body of research provides support for this viewpoint, the proposed phyletic status of Vertebrata has yet to gain widespread acceptance [17].. We review the results of recent studies in the molecular phylogeny of metazoans, comparative analysis of gene families, vertebrate-specific phylotypic stage, and body plan formation specific to vertebrates, and suggest that, based on this body of evidence, it is time for the zoological community to revisit the classification of the vertebrates.

### Molecular phylogeny

The introduction of molecular phylogeny and its application to metazoans first occured in the 1980s. The initial use of molecular phylogeny was delayed in metazoans compared with other organisms such as prokaryotes, fungi, and plants, because metazoan phylogeny had been discussed in terms of the distinct characteristic features of each taxon, including fossil records, modes of embryogenesis, and larval and adult morphology. These basic methodological approaches to metazoan classification were well-established and had a long history of providing valid insights, and thus appeared too robust to be reevaluated using other methods. However, it was soon recognized that molecular phylogeny is a very useful method for inferring relationships between metazoan taxa at the family and order levels. Nevertheless, there are a number of issues regarding the phylogenetic position of metazoan taxa at the phylum level remain, including the nature of the ctenophore ancestor of all metazoans [13, 18, 19] and the association of *Xenoturbella* with the deuterostome ancestor [20]. (The latter issue is not discussed here, as we do not consider this animal group to fall within the scope of mainstream deuterostome evolution.) Many molecular phylogenic reports have tackled the classification or taxonomy of metazoans. We discuss three examples below.

The first example is the seminal report of two major clades of protostomes: Lophotrochozoa (platyhelminths/annelids/mollusks) and Ecdysozoa (arthropods/nematodes) [13]. Protostomes are the largest group of bilaterians. The traditional view of protostome phylogeny emphasized the grade of complexity of the body plan; especially the development of the body cavity or coelom [21]. Protostomes were subdivided on the basis of the mode of body cavity formation into acoelomates (with no distinct body cavity) such as platyhelminths; pseudocoelomates (with a poorly developed body cavity) such as nematodes; and coelomates (with a distinct body cavity) such as annelids, mollusks, and arthropods. An important argument was therefore whether the presence of a metameric body plan or trochophore-like larvae was critical for the classification of eucoelomic annelids, mollusks, and arthropods. The former provided a close relationship between annelids and arthropods, whereas the latter supported the intimate relationship between annelids and mollusks. Both the report by Aguinaldo et al. [12] and that of Halanych et al. [22] influenced many zoologists. Although several later researchers (e.g., [23]) have suggested that the clade “Lophotrochozoa” should be renamed “Spiralia,” the Lophotrochozoa/Ecdysozoa classification has gradually gained acceptance. Recent comparative genomic studies suggest that ecdysozoans (arthropod–nematode clade) are a unique bilaterian group with gene families different from those of other groups, including diploblasts. (See section 2.)

The second example of the application of molecular phylogeny is the rearrangement of animal groups in relation to the phylum Annelida. Traditionally, Annelida was comprised of two major groups: Clitellata (earthworms and leeches) and Polychaeta (bristle worms). On the other hand, Sipuncula (peanut worms), Echiura (spoon worms), and Siboglinidae or Pogonophora (beard worms) were each recognized as independent phyla [24]. Recent molecular phylogeny suggests that these three taxa are also included in the larger taxon or phylum Annelida [13, 25]. Although the positions of some sub-taxa remain uncertain, this scheme has gradually been accepted in the context of a robust evolutionary history of annelids and related bilaterians. In this system, either the peanut worms and spoon worms lost body segmentation during their evolution, or the annelids obtained their segmentation pattern independently.

The third example is the taxonomic expansion of reptiles among vertebrates. Traditionally, Gnathostomata comprises six classes—Chondrichthyes, Osteichthyes, Amphibia, Reptilia, Aves, and Mammalia—although it has been suggested that Aves (birds) branched off from the reptile lineage Archaeopteryx. Recent decoding of the genomes of reptiles [26] and birds [27], as well as molecular phylogenetic analysis [28], has clearly shown that the bird clade is incorporated among different clades of reptiles. In other words, Aves is now recognized as a lineage leading to a specific group within a complex set of reptiles.

Returning to the question of the phylogenetic relationship of deuterostome taxa, what has molecular phylogeny told us of the phylogenic positions of chordates and vertebrates? An early phase of deuterostome molecular phylogeny showed a grouping of echinoderms and hemichordates [29, 30]; these are named “Ambulacraria,” as originally proposed by Metchnikoff [31]. However, these studies failed to give a clear resolution of Ambulacraria/Chordata relationship due to the problem of long branch attraction caused by the fast substitution rate of urochordate sequences in the construction of molecular phylogeny trees.

**Fig. 2:**
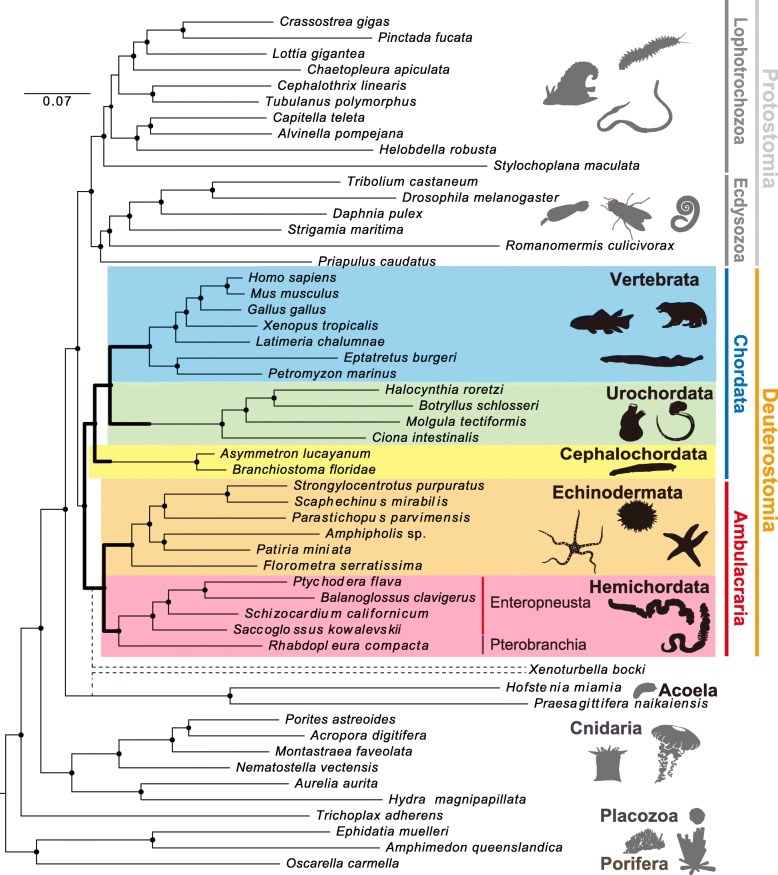
Molecular phylogeny of deuterostome taxa within the metazoan tree. Echinoderms are shown in orange, hemichordates in magenta, cephalochordates in yellow, urochordates in green, and vertebrates in blue. The maximum-likelihood tree was obtained with a supermatrix of 506,428 amino acid residues gathered from 1564 orthologous genes in 56 species (65.1% occupancy), using a Γ + LG model partitioned for each gene. Plain circles at nodes denote maximum bootstrap support. This tree clearly indicates that Deuterostomia comprises two discrete groups, Ambulacraria and Chordata (*from* [35])

In 2006, Delsuc et al. [32] performed an analysis that incorporated orthologous amino acid sequences of appendicularians and cephalochordates and demonstrated that, within the chordate clade, cephalochordates diverged first, and urochordates and vertebrates formed a sister group, as “Olfactores” (Fig. 1c) This relationship has been supported by further analyses that include different taxa and larger quantities of higher-quality molecular data [33, 34]. Debates on the evolutionary scenarios of sedimentary and free-living ancestors are now likely resolved: Chordate ancestors were free-living, like lancelets [17]. Figure 2 is a molecular phylogenetic diagram that pays particular attention to deuterostome relationships [35]. The tree was constructed by comparing the positions of ~ 500,000 amino acids of 1565 families with single-copy orthologs present in 53 metazoan species with 30 sequenced genomes; presence–absence characters for introns and coding indels were also incorporated. This and other previous molecular phylogenetic studies have unambiguously demonstrated (1) the division of deuterostomes into two major groups—ambulacrarians and chordates; and (2) the divergence of cephalochordates first among the chordate lineages. On the basis of a relaxed molecular clock that incorporates data from fossil records and rates of amino acid substitution, the divergence time of deuterostomes and protostomes was estimated to be ~ 670 Mya; that of ambulacrarians and chordates ~ 660 Mya; that of echinoderms and hemichordates among the ambulacrarians was ~ 600 Mya, and that of the three chordate groups ~ 650 Mya [35]. It is thus likely that chordates diverged earlier than, or at least at a similar time to, ambulacrarians. If we accept t that Echinodermata and Hemichordata are two phyla of the higher clade Ambulacraria, then it might also be accepted that Chordata is another higher clade that comprises three phyla: Cephalochordata, Urochordata, and Vertebrata. In other words, Vertebrata may be more correctly described as a phylum, not a subphylum of the phylum Chordata.

### Whole-genome duplication and gene-family expansion

Vertebrates experienced a two-round whole-genome duplication (2R-WGD) during their evolution. The vertebrate-specific 2R-WGD has been supported by a great variety of evidence, including the existence of the Hox cluster, as discussed below. Whole-genome duplication also occurred in some ecdysozoan species, including the Atlantic horseshoe crab [36], house spider [37], and hexapods [38]. In general, WGD has been considered a major force of genome evolution that promotes animal diversity. However, it has been pointed out that WGD in these arthropods did not always lead to developmental and morphological diversity within groups. In contrast, vertebrate WGD may cause a supra-ordinal expansion of gene families, which is highly likely to represent the evolutionary force behind the complexity and diversity of vertebrate body plans. (See sections 3 and 4.)

#### Hox clusters

Vertebrate-specific 2R-WGD has been exemplified by various genes and gene families, of which the Hox cluster is the best-known example. In most groups of protostomes and deuterostomes studied at high taxonomic levels to date, Hox genes (encoding homeodomain-containing transcription factors) are clustered in the same genomic region, known as the Hox cluster. The Hox cluster shows spatial and temporal collinearity. That is, the expression patterns of Hox genes reflect their positions in the cluster. Genes at the 3′ end are expressed in, and pattern, the anterior end of the embryo, whereas genes at the 5′ end pattern the more posterior body parts (spatial collinearity). Moreover, gene position in the cluster also determines the time of onset of expression, with 3′-end genes expressed in earlier developmental stages than those at the 5′ end (temporal collinearity). As a result, Hox genes are eventually expressed in a nested manner along the anterior–posterior axis of the animal body, resulting in a Hox code that bestows differential structural identity. (See section 4.)

Recent studies have revealed the organization of deuterostome Hox clusters— especially in echinoderms and hemichordates—and have thus shed more light on vertebrate-specific duplication of the Hox cluster in deuterostome taxa with shared common ancestors. Below, we discuss recent studies of the Hox cluster in cephalochordates, hemichordates, echinoderms, urochordates, and vertebrates.

**Fig. 3:**
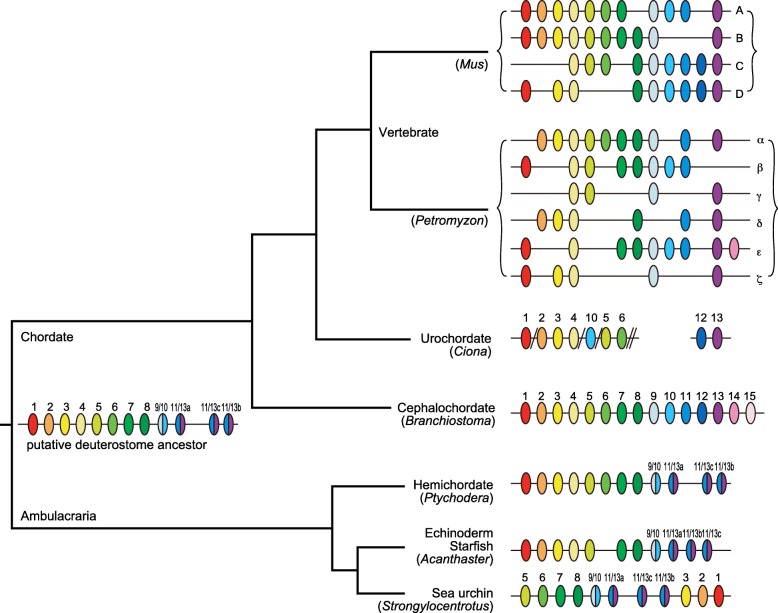
Hox-cluster gene organization in deuterostomes. Colored ovals indicate Hox genes. Genes of smaller paralogous subgroup numbers are to the left and those of larger numbers are to the right. A putative chordate ancestor may have possessed a single Hox gene cluster with ~ 13 genes. This would have been conserved in hemichordates and cephalochordates, although cephalochordates must have undergone duplication of the posterior-most genes. Echinoderms most likely lost *Hox6*. In urochordates, the Hox cluster was reorganized; in *Ciona intestinalis*, Hox genes are mapped on two chromosomes and the putative gene order is shown for *Hox2* to *4* and *Hox5* and *6.* Jawed vertebrates, except teleosts, have four Hox gene clusters (HoxA to HoxD from top to bottom), whereas the sea lamprey contains six Hox gene clusters (Hox-α to Hox-ζ). (*References are in the text*)

The Hox cluster of cephalochordates has been studied extensively, as this taxon represents a key phylogenetic position for deducing the ancestral condition of chordates, and is a valuable out-group for evolutionary studies of vertebrates [39]. Cephalochordates possess the most prototypical Hox cluster identified so far in deuterostomes: the Floridian amphioxus *Branchiostoma floridae* contains a typical 13 genes, including three anterior (*Hox1* to *3*), six middle (*Hox4* to *10*), and three posterior (*Hox11* to *13*) genes (Fig. 3). The cluster also contains *Hox 14* and *15*, which represents the largest gene content for a Hox cluster hitherto reported, spanning a genomic stretch of ~ 470 kb, all in the same transcriptional orientation. The cluster has not suffered any rearrangements since the cephalochordates split from their chordate ancestor. However, discussion continues as to whether *Hox14* is shared by basal groups of vertebrates, and whether *Hox15* is a true member of the cluster [40, 41].

Among ambulacrarians, hemichordates are thought to retain more features of the last common ancestor than echinoderms [17, 42]. A recent study identified the presence of a single Hox cluster in the genomes of two enteropneusts (acorn worms), *Saccoglossus kowalevskii* and *Ptychodera flava* (Fig. 3) [43]. The hemichordate Hox cluster reflects a prototypical organization among deuterostomes, showing an organization with 12 Hox genes arrayed in ~ 500 kb, all with the same transcriptional orientation, except for the terminal pair of ambulacrarian-specific posterior Hox genes, *AmbPb* and *AmbPc* (previously named *Hox11/13b* and *Hox11/13c*, respectively [43] (Fig. 3). The conservation of echinoderm Hox clusters has also been disclosed recently. The Hox cluster of the sea urchin, *Strongylocentrotus purpuratus*, is a single cluster of about 600 kb that contains 11 Hox genes (*Hox4* is missing) [44] (Fig. 3). It also appears to have undergone re-ordering, as *Hox1*–*3* are located near the posterior end of the cluster (Fig. 3). In addition, sea urchin Hox genes are expressed not during embryogenesis but during juvenile development. These data suggest that this Hox shuffling is associated with echinoderm-specific pentameric symmetry, although the function of these rearranged genes remains to be elucidated. However, the crown-of-thorns starfish, *Acanthaster planci*, has an organized Hox cluster of 11 genes, in which *Hox6* is missing (Fig. 3) [45]. Because this starfish with pentameric symmetry retains an organized Hox cluster, the relationship between the echinoderm Hox rearrangement and pentameric symmetry requires further investigation.

As discussed above, Hox genes are conserved in well-organized clusters in both ambulacrarians and cephalochordates. The urochordates, however, represent an interesting exception. Urochordate genomes are highly divergent. For example, *Ciona intestinalis* possesses an atypically organized set of Hox genes [46–48]. The Hox cluster is divided into two groups located on different chromosomes [49]: *Hox1* to *6* and *10* on chromosome 1 and *Hox12* and *13* on chromosome 7 (Fig. 3). In addition, *Hox7* to *9* and *11* are absent in all ascidians sequenced so far. Nevertheless, collinearity seems somehow to have been retained in the *Ciona* Hox cluster [47].

In contrast to the single Hox cluster of invertebrate deuterostomes, jawed vertebrates contain four Hox clusters; because of 2R-WGD, the Hox clusters of vertebrates have increased to four paralogous groups, HoxA to HoxD (Fig. 3). If the Hox cluster of the last common ancestor consisted of 12 or 13 genes, 2R-WGD would imply the presence of 48 or 52 homeobox genes in vertebrates. In all such events, however, duplication of the Hox cluster was followed by the loss of various Hox gene, resulting in unique combinations of Hox genes in different groups, which can serve an identifying function akin to that of bar codes (a “genomic Hox-bar code”) [50]. Comparison of the Hox inventories of different tetrapods has shown that there was a tetrapod ancestral condition of up to 41 Hox genes [51] and an amniote ancestral condition of 40 Hox genes (after the loss of *HoxC1*), the full set of which is retained only by the green anole (*Anolis carolinensis*). Mammals and chickens have lost *HoxC3* independently. Although the western clawed frog (*Xenopus tropicalis*) has 38 Hox genes, the amphibian ancestor probably had 40 genes, after losing *HoxD12*.

Recent studies of Hox clusters in vertebrates have revealed more complex histories of WGD in vertebrates. First, the two main groups of gnathostomes are the chondrichthyans (cartilaginous fishes) and the osteichthyans (bony fishes). In contrast to the apparently complete loss of the HoxC cluster in elasmobranchs [52], teleosts are likely to have experienced an additional round of duplication (third WGD); the Atlantic salmon, *Salmo salar*, and the rainbow trout, *Oncorhynchus mykiss*, have 13 Hox clusters, arising from a salmonid-specific fourth round of WGD [52]. *Salmo* has 118 Hox genes plus eight pseudogenes [53]—the largest Hox repertoire reported to date.

The organization of the Hox cluster has been examined in cyclostomes (agnathans, or jawless vertebrates), yielding interesting results that shed light on the question of when the first and second round of WGD occurred. Cyclostomes are composed of two different groups, lampreys and hagfishes. In the case of the hagfish, Stadler et al. [54], using degenerate PCR, reported the presence of up to 33 Hox genes—fewer than expected—and Pascual-Anaya et al. [55] showed the conservation of temporal collinearity, as seen in jawed vertebrates. On the other hand, the sea lamprey *Petromyzon marinus* exhibits a unique phenomenon known as programmed genome rearrangement, in which, during embryogenesis, some portions of the genome are abbreviated such that the somatic cells retain only a portion of the genome originally retained in germ cells [56]. Recent decoding of the *Petromyzon* germ-cell genome by Smith et al. [57] revealed the presence of six Hox clusters in the genome (Hox-α, Hox-β, Hox-γ, Hox-δ, Hox-ε, and Hox-ζ) (Fig. 3). Smith et al. discussed the important role of chromosomal-level duplication and the duplication of large-scale genomic regions in the establishment of Hox clusters on different chromosomes. It is therefore now highly likely that 2R-WGD occurred in the ancestor(s) of all vertebrate groups. This likelihood offers support for vertebrates as a discrete animal taxon that is distinct from any of the notochordal invertebrate taxa.

#### (b) Expansion of gene families

**Fig. 4:**
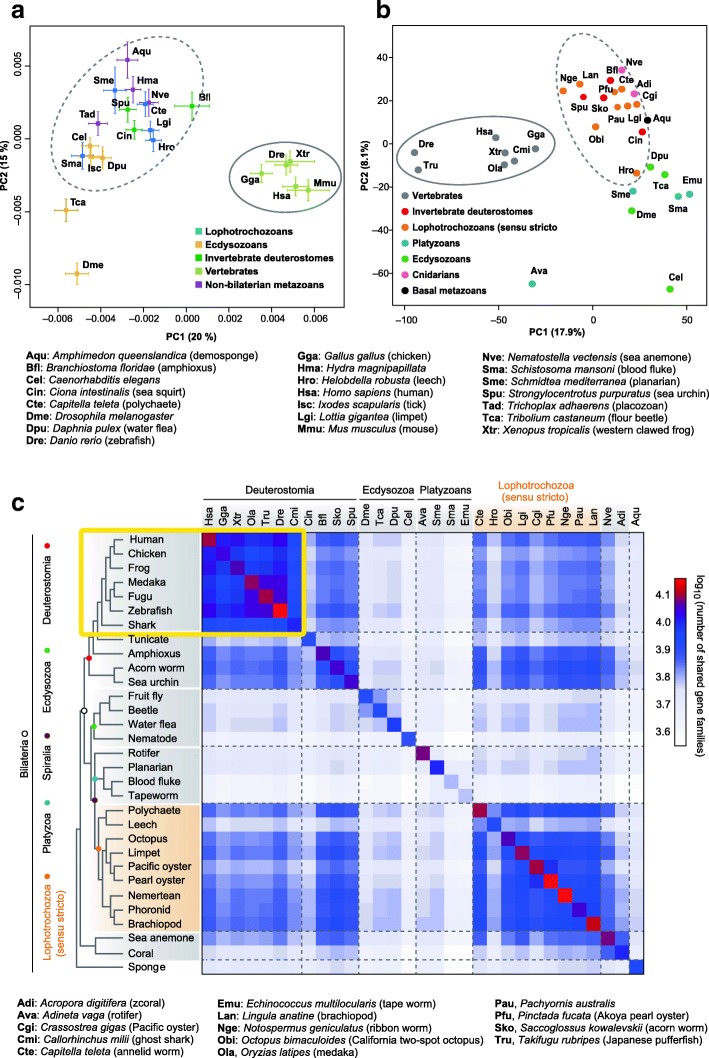
Clustering of metazoan gene family composition. Shown are the results of two independent analyses performed by (**a**) Simakov et al. [35] and (**b, c**) Luo et al. [61]. The first two principal components are displayed. **a** Principal component (PC) analysis of annotated gene functions. At least three clusters are evident, namely a vertebrate cluster (far right; solid-line circle); a non-bilaterian metazoan, invertebrate deuterostome, or spiralian cluster (center, top; dashed-line circle), and an ecdysozoan group (lower left). *Drosophila* and *Tribolium* (lower left) are outliers. **b** PC analysis of PANTHER gene family sizes. Invertebrate deuterostomes (Bfl, Sko, and Spu) cluster with lophotrochozoans (dashed-line circle). Solid-line circle denotes the clustering of vertebrates. In addition to the metazoan species analyzed in (**a**), the following species were included in the analysis. **c** Matrix of shared gene families among selected metazoans. The cladogram on the left is based on phylogenetic positions inferred from this study. Dashed lines separate the major clades. Note that tunicates (Cin) and leeches (Hro) share fewer genes with other bilaterians, probably because of their relatively high evolutionary rates and gene loss in each lineage

Vertebrates are characterized by various morphological, developmental, and physiological features. (See sections 3 and 4.) These include the neural crest and placodes that are involved in the formation of “new head” structures [58]; a complex central nervous system; jaws including pharyngeal gill structures; bone; an adaptive immune system; and a hormonal system associated with the hypothalamus, pituitary, and gonad [59]. Here, we do not discuss the evolution of these features; it seems reasonable that their evolution occurred through the expansion or diversification (or both) of gene families as a result of 2R-WGD. Recent decoding of the genomes of various animal groups has made it possible to compare gene family compositions among deuterostomes as well as protostomes and diploblasts. Custom clustering analysis revealing the numbers of gene families shared by deuterostomes (8716), ambulacrarians (9892), and chordates (9957) implies the presence of at least 8716 families of homologous genes in the deuterostome ancestor [35]. Analyses of these gene families have demonstrated the remarkable complexity of the vertebrate gene family (Fig. 4); this provides further evidence for vertebrates as a distinct and independent animal taxon.

So far at least three genome-decoding studies have analyzed the evolutionary changes in the content of gene families in metazoans [35, 60, 61]. The first analysis included the genomes of three lophotrochozoan species (one mollusk and two annelids) [60]; the second, two hemichordates [35]; and the third, nemertean, phoronid, and brachiopod genomes [61]. Figure 4a and b illustrate the work of Simakov et al. [60] and Luo et al. [61], respectively. Although different numbers of metazoan species are included, both results clearly indicate the independent and discrete clustering of vertebrate species (solid circles) from invertebrate species (dashed circles). This suggests that vertebrates differ distinctly from invertebrates in the constituents of gene families. These analyses show the affinity of deuterostomes and spiralians (dashed circles). Ecdysozoans, in contrast, were clustered or scattered apart from other metazoans (Fig. 4a and b), suggesting the specificity of this protostome group.

Additional hierarchical clustering analysis of gene families shared exclusively among metazoans clearly indicates a vertebrate-specific cluster (top-left corner of Fig. 4c, enclosed by yellow box) [61]. This figure also shows (1) clustering of the gene repertoire in deuterostomes (gapped by tunicates; upper right); (2) spiralian species (lower right) with affinity to cnidarians; and (3) an ecdysozoan-specific cluster (middle), suggesting that ecdysozoans diversified independently of other metazoans. In summary, as indicated by recent studies of molecular phylogeny and comparative genomics, vertebrates are unique among bilaterians and distinct from invertebrates. Taking these findings into consideration, it is evident that the prospective phylum Vertebrata is the first clearly classifiable animal phylum.

### Vertebrate-specific phylotypic period

An animal phylum is generally defined as a monophyletic group of animals that share the same body plan (a set of basic anatomical features shared by animals of a certain lineage). A key problem in defining phyla thus lies in the difficulty in identifying distinct body plans among different animal groups; the situation becomes even more challenging when extinct species are taken into consideration [62]. In this sense, defining phyla is analogous to finding boundaries between continuous mountains, and this is the logic we have followed so far in the previous sections. Given that the boundaries should now be settled between, for example, hemichordates and echinoderms, it should now also be possible to settle on the other, comparable, boundaries between the three major chordate groups. In addition to the discussion above, another potential support for “phylum Vertebrata” has recently been suggested by the results of a comparative transcriptomic study of chordate embryos [63].

**Fig. 5:**
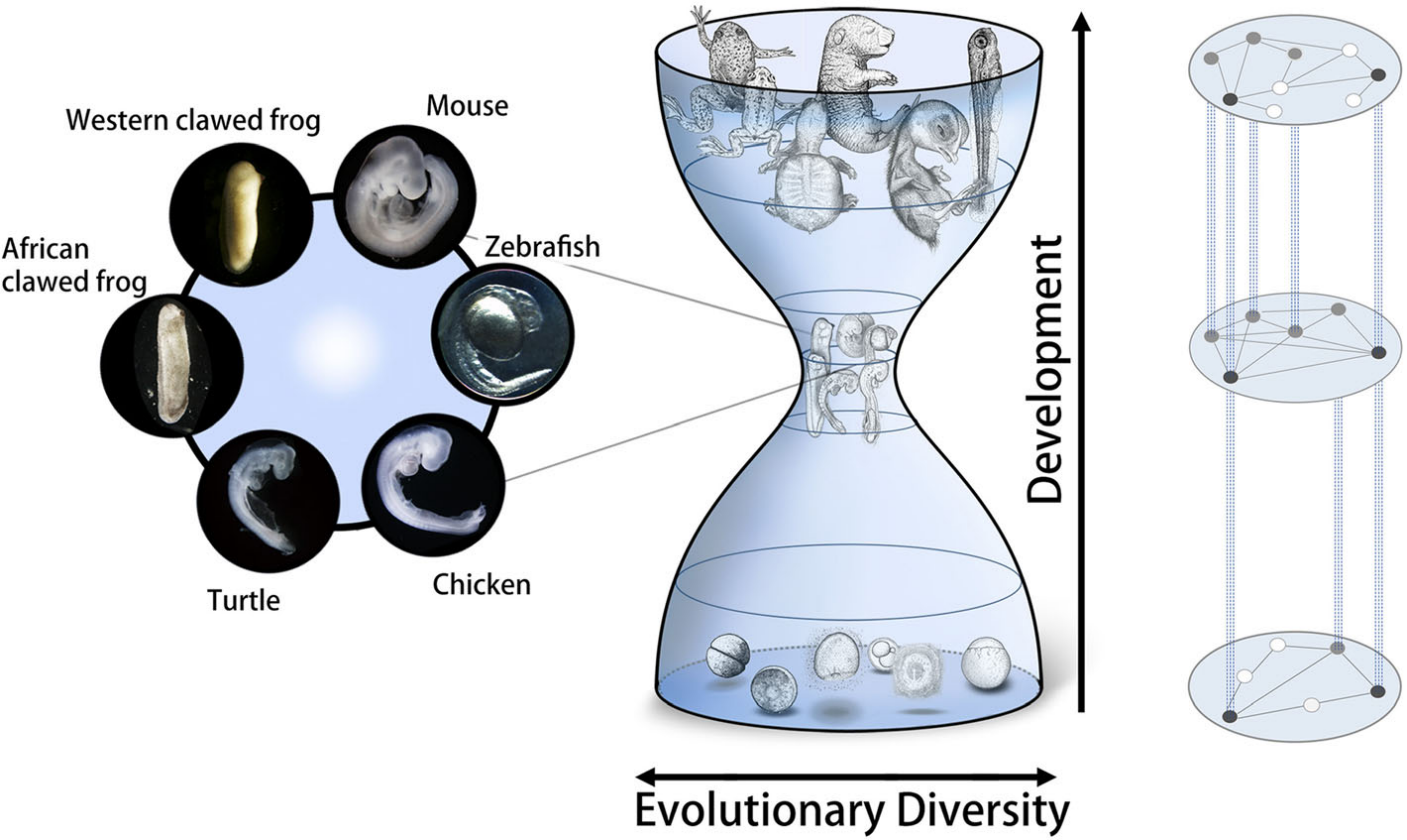
The developmental hourglass model and embryos representative of phylotypic periods in vertebrates. In the developmental hourglass model (middle, originally proposed by Duboule [64]), embryogenesis proceeds from the bottom to the top, and evolutionary divergence becomes minimal at the mid-embryonic, organogenesis phase. The conserved mid-embryonic phase has been predicted to define the body plan for each animal phylum [64] and has therefore been named the phylotypic satage [64, 65, 66, 181]. However, further studies are required to fully verify this hypothesis, and a recent study indicated that the hypothesis may be better applied to vertebrates than to chordates [63]. Embryos representative of most conserved vertebrate stages are shown at the left. Curiously, these stages can also be identified as the most conserved stages when comparing groups of species smaller than at the vertebrate level. No consensus has been reached on the mechanism of this mid-embryonic conservation, but two independent studies have implied possible contributions by developmental constraints [63, 74]. In other words, extensive reuse of the same genetic machinery could have imposed limitations on the evolutionary diversification process through pleiotropic constraint (right, modified from Hu et al. [63]). In each developmental stage, grey and black dots represent genetic components that are pleiotropically expressed in other stages and are shared (blue vertical lines) by multiple developmental processes

The study tested the phylotype hypothesis of the developmental hourglass model [64] by using chordate species [63]. According to the developmental hourglass model, the mid-embryonic, organogenesis period is the phase that is most conserved through embryogenesis (Fig. 5), and this phylotypic period defines the body plan for each animal phylum (phylotype hypothesis, [64–66]. Although recent transcriptomic studies have supported the presence of hourglass-like, mid-embryonic conservation patterns in a variety of animal groups, including vertebrates [67–71], *Drosophila* species [72], *Caenorhabditis* species [73, 74] and mollusks [75], the range of species compared was much narrower than at the phylum level, and it remains unclear whether the hourglass model holds true for each animal phylum. Specifically, for vertebrate species, the transcriptomically identified, most conserved mid-embryonic stages (Fig. 5, left) did have morphological elements that were shared among chordates, such as a dorsal nerve cord and notochord. However, none of the previous studies covered non-vertebrate chordates to see whether these stages could still be identified as the most chordate-conserved stages. Levin et al. [76], in cross-phylum comparisons using 10 animals from different phyla (single species from poriferans, cnidarians, nematodes, arthropods, chordates, echinoderms, annelids, platyhelminths, ctenophores, and tardigrades), observed no mid-embryonic conservations, although Dunn et al. [77] raised methodological concerns regarding their study.

In a comparative transcriptome study reported by Hu et al. [63], eight chordate species, including two non-vertebrate chordates, were analyzed. Mid-embryonic conservation was robustly supported among the vertebrate species, as in previous studies, but the results for the chordates were not concordant among the methods of transcriptome comparison. In short, analysis of strictly conserved 1:1 orthologous genes (1704 in total) in the eight chordate species revealed that the mid-embryonic phase was transcriptomically conserved (including in the two non-vertebrates: *C. intestinalis* at around stages 22 to 27 and *B. floridae* at around the neurula to early larval stages); the identified stages showed a set of chordate body plans, namely the notochord and dorsal nerve cord. However, when paralogous genes and genes lost in certain species were taken into consideration in the transcriptomic comparison, mid-embryonic conservation was not observed among chordate species. These results imply that conserved genes retained since chordate common ancestors are still expressed in the mid-embryonic phase of *C. intestinalis* and amphioxus, but that the overall degree of conservation becomes obscure when lost and duplicated genes are taken into consideration. The conservation boundary between vertebrates and non-vertebrate chordates appears to coincide well with both the 2R-WGD that occurred in the vertebrate lineage, as well as with the morphological differences between vertebrates and non-vertebrate chordates (e.g., the pharyngeal arch in vertebrates and gill slit in *Ciona*).

Interestingly, the conserved stages found among vertebrates largely overlapped with those conserved in smaller groups, such as tetrapods and amniotes, and even among *Xenopus* frogs or between turtle and chicken. This persistent conservation [78, 79] suggests that these phases of organogenesis in vertebrates remained targets of conservation since the emergence of their common ancestors. Put differently, this provides an alternative way of defining a phylum by grouping species that show persistent conservation of the same developmental phase, as the highly conserved mid-embryonic phase represents the body plan of a given group. Hu et al. [63] imply that vertebrates can better be grouped as a phylum than under the chordate phylum. We still do not know whether this can be applied to other animal groups, but independent studies have reported mid-embryonic conservation in species other than chordates in groups much smaller than a phylum (*Drosophila* genus [72], and *Caenorhabditis* species and their experimental lineages [73, 74]).

Further studies are required to fully test the idea of defining body plans by persistent conservation of the mid-embryonic phase, because early-diverged vertebrates, such as the lamprey and hagfish, have not yet been included in such investigations. Furthermore, even if pharyngula embryos show persistent conservation within vertebrate lineages, we do not know how that conservation took place. Although the contributions of negative and positive selection per se appear insufficient to explain mid-embryonic conservation [74, 80]), contributions by other mechanisms, such as developmental burden [74] and pleiotropic constraint [72] (Fig. 5, right) are being suggested by experimental studies. Investigating how animals appear to have broken the rule of body plan could also help us to better understand this question [81]. Moreover, identification of the general mechanism behind the hourglass-like conservation model in future studies would be a great help in better understanding the body plans found in various animal groups and in classifying phyla.

### Body plan and embryogenesis

Vertebrates are characterized by a number of specific, derived features that are not found in non-vertebrate chordates or invertebrates. Examples include a well-developed cranium (or head), vertebrae, paired eyes accompanied by extrinsic eye muscles, median fins, a ventrally opening mouth, and a postanal tail (reviewed in [82–84]). The presence of trunk-specific myotomes could also be vertebrate-specific. Other basic characters, including a dorsal neural tube, pharynx with gill pores (or slits), and notochord, are more widely distributed among chordates. Importantly, the gill slits or gill pores are also shared by ambulacrarians, representing a synapomorphy for deuterostomes, not for chordates [35, 83].

Embryologically, the vertebrate pharyngula—the vertebrate embryo at about the organogenetic period—has been regarded as exhibiting the most conserved embryonic pattern (see below), and the development of the vertebrate body plan is thought to be laid upon the establishment of this embryonic pattern (see [64, 85]). The pharyngula is characterized by the presence of ectomesenchyme-containing pharyngeal arches in the ventral head; these are also unique in vertebrates (reviewed in [86–88]; for the development and evolution of the pharyngeal arches, see [89]).

In the pharyngula, we find not only specific sets of organs and embryonic primordia, but also vertebrate-specific integration and connection of elements, each of which occupies its specific and equivalent position in the body and shows a constrained linkage with other elements. For this constraint, a specific set of morphological homologies becomes recognizable. This anatomical integration—the body plan mentioned above—becomes first visible at the phylotypic period when the phylum-specific developmental constraints become embodied (see [90, 91] for reviews). The interesting question, therefore, is whether there is a chordate-specific body plan in terms of developmental and anatomical patterns, and whether it can be derived through modification of the chordate body plan. We need also to determine whether the vertebrate body plan should be viewed as merely a variation of the chordate body plan, or whether it is distinct enough to establish an independent phylum.

#### The vertebrate-specific pattern

On the basis of the presence of the notochord, Kowalevsky recognized close affinity among tunicates, amphioxus, and vertebrates [9, 10]. Ernst Haeckel was strongly influenced by this theory, and he classified amphioxus as a vertebrate [92]. To distinguish the true vertebrates (as defined at the time) from the amphioxus, he created a subcategory, the craniates (animals with a head), as opposed to the acraniate amphioxus. Thus, craniates were once an in-group of “vertebrates,” defined by Haeckel, which were defined similarly to today’s Chordata. Subsequently, the name Vertebrata began to be applied only to animals with backbones, as is currently understood (reviewed in [17]). Until recently, amphioxus and hagfish were technically called “invertebrates” [93–95]. To stress the similarity between hagfish and other vertebrates—especially to stress the possession of an overt head—the name Craniata was secondarily applied to mean “vertebrates plus hagfish.” At that point, therefore, vertebrates were defined as an in-group of craniates [93–95].

Recently, molecular analyses have unanimously supported the monophyly of cyclostomes (lampreys and hagfishes), and the taxonomic name Craniata has become invalid [96–102] (reviewed in [103]). The morphological definition of vertebrates largely relies on an understanding of hagfish, of course: Besides the absence of vertebral elements, the anatomy of the hagfish is very different from that of other vertebrates.

Since 2007, our knowledge about hagfish development has been greatly improved, and it is now known that the hagfish develops vestigial vertebral elements, and that the developmental patterns of this animal and the lamprey are very strikingly similar during the early pharyngula period. These animals develop cyclostome-specific anatomical patterns not shared by modern jawed vertebrates, with complicated patterns of chondrocrania that are perfectly comparable between the two animals [104, 105] (reviewed in [103]). The morphological difference between the crown gnathostomes and cyclostomes stems primarily from the transition from single (cyclostomes) to double (crown gnathostomes) nostrils that resulted in a modified distribution of the craniofacial ectomesenchyme [106, 107]. Otherwise, however, the embryonic patterns of cyclostomes and crown gnathostomes are very similar. In the pharyngula (which is specific to vertebrates, including the cyclostomes), a set of morphological traits is consistently found across species; this includes somites with somitomeric elements, head mesoderm, neural-crest-derived ectomesenchyme, pharyngeal arches and associated branchiomeric structures, and placodes and their derivatives [108, 109].

Vertebrates also have a unique and specific body plan not shared by other chordates. It is noteworthy that von Baer (1828) called the pharyngula the *Haupttyp* (meaning ‘major type’) [85], implying that the conceptual archetypal pattern of vertebrates is embodied therein, not as an idealistic pattern but in actual embryonic morphology. The spirit of this nomenclature is that the most basic set of structures for defining vertebrates appears at this particular stage—the phylum-defining stage—emerging at a specific period in the developmental timetable and followed by more specialized stages that define lower ranks of taxa. Von Baer, who did not believe in evolution, thought that developmental patterns reflected the nested relationships of hierarchical taxa. To evaluate the vertebrate phylotype, therefore, it is necessary to characterize the vertebrate pharyngula from the embryological and morphological perspectives. Importantly, Haeckel included amphioxus in vertebrates owing to the resemblance of adult anatomy, not embryonic patterns, to the idealized pattern of vertebrates; amphioxus embryos were compared mainly with cnidarians [108]. Although tunicates are phylogenetically regarded as the lineage closest to the vertebrates (see above), acraniates (amphioxus) are generally thought to represent the best proxy for understanding the origin of the vertebrate body plan, because tunicate developmental patterns are secondarily modified [109]. Therefore, in this section we examine amphioxus as a model for examining the uniqueness of the vertebrate body plan.

As noted above, the definition of the group Vertebrata is tightly linked with the evolutionary origin of the unique head (or cranium), which is absent from amphioxus, and characterizes most clearly the morphological quality and evolutionary origin of the vertebrate body plan per se. Curiously, the vertebrate head develops on the basis of vertebrate-specific cell lineages—the neural crest, along with ectodermal placodes and non-segmented head mesoderm, none of which is found in non-vertebrate chordates [56, 110]. The evolutionary origin of the neural crest cell lineage has been studied intensively for the past decade, because its acquisition is expected to yield indirect insights into the origin of the head [111–118]). Simultaneously, it has been stressed by Linda Holland and her colleagues that the amphioxus has primarily a rostral end identical to that in vertebrates, in terms of developmental regulatory gene expression profiles; nothing had to be added in the rostral end of the hypothetical ancestor to acquire the vertebrate head [119–123] (also see [124]). We revisit this issue below in a different context of vertebrate morphogenesis.

It seems likely that the evolution of the vertebrate head involved radical changes in developmental programs and the rewiring of associated gene regulatory networks (see [115]). However, the identification of truly vertebrate-specific features is not always straightforward, especially because the precursors of traits can often be found in non-vertebrate chordates [105, 111]. (For a similar argument regarding evolutionary precursors, see [124].)

#### Head

The head mesoderm is unique in vertebrates in that it arises as a non-segmented mesoderm. The morphological and evolutionary origin of the head mesoderm is enigmatic, and historically this question is tightly linked to the idea of head segmentation [125–127]. Whether or not the vertebrate head mesoderm is homologous to the rostral somite (or somites) of amphioxus, the lack of overt mesodermal segments in the vertebrate head provides the vertebrate-specific embryonic environment and distinct morphology of the cranial nerves in these animals ([125]; also see [126]). Unlike the somite-dependent somitomeric organization of the spinal nerves, pharyngeal-arch-associated nerves (branchiomeric nerves) develop on rhombomeres (segmental bulges of the hindbrain) and are distally associated with the epibranchial placode, exhibiting metameric patterns that collectively mirror the branchiomeric segmental pattern of vertebrates. Thus, the vertebrate body plan is characterized by the possession of dual (or triple, including enigmatic neuromeres) metamerism that is most evident in the morphology of the peripheral nervous system [125, 127, 128].

The vertebrate head mesoderm is the source of extrinsic eye muscles and the primary neurocranium that encapsulates the enlarged brain [108, 129, 130]. (For development of the extrinsic eye muscles, see [131].) The latter skeletal element is unsegmented, as opposed to the ventral moiety of the cranium, the viscerocranium, which is derived from the neural crest cells in the segmented pharyngeal arches ([130, 132], but see [125, 133, 134]). Part of the viscerocranium is secondarily incorporated into the neurocranium to form its rostral part in jawed vertebrates (reviewed in [108, 135]). Thus, the vertebrate cranium has two major components, derived from multiple cell lineages and differentiating into various types of skeletal tissues ([136]; also see [137, 138]).

Unlike vertebrates, amphioxus shows striking asymmetry in both adult morphology and embryonic developmental patterns. For example, its mouth opens on the left side of the embryo, but shifts secondarily to a pseudo-symmetrical position in the adult (asymmetry is still evident in the innervation pattern of the oral hood). Hatschek’s pit—the suggested homolog of the adenohypophysis—also develops on the left side, and in the adult, myotomes on the left and right sides show a staggered pattern of arrangement along the body axis [139–141]. Amphioxus does not possess a cranium of any sort, nor any skeletal tissues comparable to those in vertebrates. It also develops pharyngeal pores—on the left side only at first—which secondarily become bilaterally paired, each pore being duplicated anteroposteriorly, to result in numerous gill slits [139]. (Also see [140].) These pores never penetrate to open onto the surface in the adult, because a coelom-like structure lined by ectoderm—the peribranchial coelom—secondarily arises by the ventral growth of the left and right atrial folds, which fuse together in the ventral midline leaving a posterior opening, the atriopore. Only early in its development (48 h post-fertilization), therefore, does the amphioxus larva show externally opened pharyngeal pores, and only in the rostral-most part of the pharynx.

Somites grow ventrally into the abovementioned secondary body wall; therefore, on the surface of the amphioxus body, only a single metamerism is apparent externally [139, 141]. Thus, the vertebrate-like configuration of the pharyngeal pore is found only at the early phase of pharyngeal development, in the rostral-most part of the pharynx. Importantly, all the peripheral nerves pass between the adjacent myotomes—reminiscent of the cyclostome spinal nerves in part. They are regarded as somitomeric nerves as far as their morphology is concerned. (For the functional properties of the nerves, see [142]).

The morphological pattern of the vertebrate head cannot be derived from the amphioxus-like condition. Typically, the position of the mouth is one of the most enigmatic elements to understand. In vertebrates, the mouth arises by the perforation of the oropharyngeal membrane, a composite of pharyngeal endoderm and stomodeal ectoderm, located in the ventral midline of the head ectoderm. This feature, however, is not universal among chordates. Kaji et al. [143] have recently suggested that the amphioxus mouth, which opens on the left side of the body, is very similar to the external duct of the mesodermal coelom as found in echinoderm auricularian larvae. Histological observation and gene expression patterns are consistent with this interpretation [143].

#### Head–trunk interface and neural crest cells

**Fig. 6:**
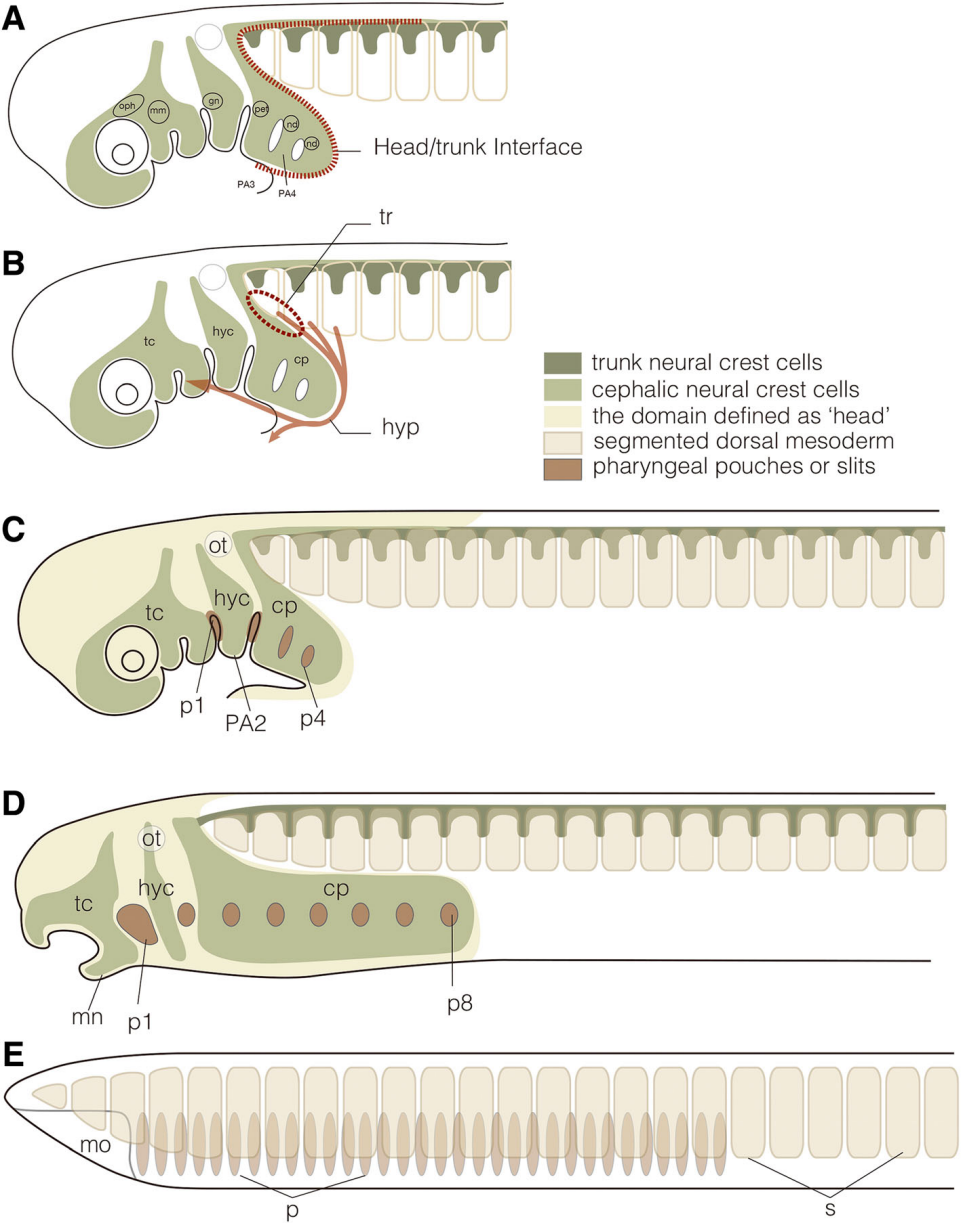
Head–trunk interface. **a** to **c**. Schematic representation of the head and trunk in the pharyngula of modern jawed vertebrates, as defined by the migratory/distribution patterns of neural crest cells (NCCs). In the head of the vertebrate pharyngula, NCCs form extensive ectomesenchyme with three major NCC populations, called trigeminal (tc), hyoid (hyc) and circumpharyngeal crest cells (cp), filling the frontonasal region and pharyngeal arches, defining the vertebrate head (yellowish region in C), as opposed to the posterior domain occupied by somites and the lateral plate. In the trunk, NCCs are segmented primarily into somitomeric streams by the presence of somites (dark green). Between the two distinct groups of NCC populations is found an S-shaped interface (red broken line). Circles denote placodes for cranial nerves (oph, ophthalmic placode; mm, maxillomandibular placode; gn, geniculate placode; pet, petrosal placode; nd, nodose placodes). In B, the position of trapezius muscle development (tr) and pathway of the hypobranchial muscle (the hypoglossal cord: hyp) are shown along the head–trunk interface. **d**. Schematization of an early lamprey larva. Note that the head and trunk can be defined in this animal as a vertebrate-specific feature. **e**. Comparison with schematized amphioxus. The mesoderm of this animal is entirely segmented into somite-like structures, but there is no overt lateral plate. Because the pharynx is located medial, not ventral, to the body wall, the head–trunk interface cannot be defined in this animal. mn, mandibular arch; mo, mouth; ot, otic vesicle; p, pharyngeal pores in amphioxus; p1 to 8, pharyngeal pouches; PA2 to 4, pharyngeal arches; s, somites

In the vertebrate pharyngula, trunk somites are restricted to posterior to the otic vesicle; rostral to the vesicle there is non-segmental head mesoderm (Fig. 6a-d). The cephalic neural crest cells migrate along a pathway called the dorsoventral pathway, which is available only at the somite-free levels. Ventrally, the cephalic crest cells are distributed in each pharyngeal arch, thereby forming an extensive ectomesenchyme as the source of cranial skeletal tissues and other types of connective tissues.

In the trunk of the pharyngula, the neural crest cells are segmented into a metameric pattern by the presence of somites (Fig. 6a-c). In amniotes, in which the behavior of the neural crest cells has been extensively studied, the crest cells are allowed to pass through only the rostral half of a somite, where part of the crest cell population will differentiate into dorsal root ganglia. This trunk-specific pathway is called the ventrolateral pathway, and it mirrors the morphological patterns of the spinal nerves and sympathetic nervous system.

**Fig. 7:**
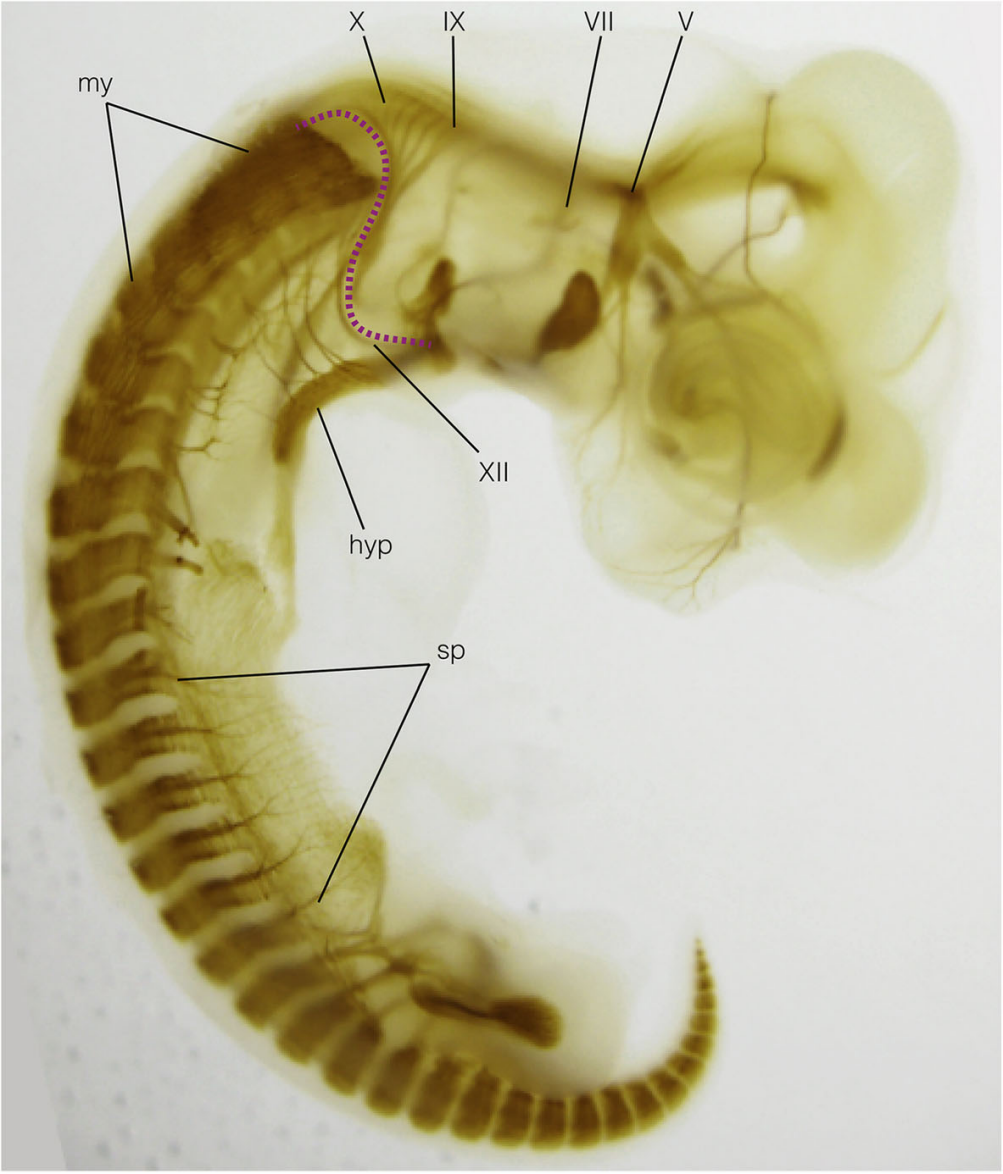
Late pharyngula of the Chinese soft-shelled turtle, *Pelodiscus sinensis*, immunostained to show peripheral nervous system and muscle primordia. The head–trunk interface is shown by the magenta broken line, delineating the spinal and branchiomeric nerves from each other. Note also that the myotomes are restricted to the trunk region of the embryo. Hyp, hypobranchial muscle anlage; IX, glossopharyngeal nerve; my, myotomes; sp., spinal nerves; V, trigeminal nerve; VII, facial nerve; X, vagus nerve; XII, hypoglossal nerve

In the postoptic region, there is an intermediate domain between the head and trunk of the pharyngula; this domain is called the head–trunk interface (Figs. 6 and 7). Because the rostral-most somites (suprapharyngeal somites) and caudal (postoptic) pharyngeal arches overlap each other dorsoventrally, at the level caudal to the otic vesicle the head- and trunk-like embryonic environments overlap to form an S-shaped interface. In this domain, the crest cells are distributed in a complex pattern. Some of the postoptic crest–derived cells behave as trunk crest cells, forming vestigial dorsal root ganglia (Froriep’s ganglia) associated with the developing hypoglossal nerve, a bundle of secondarily modified spinal nerves [141, 142]. Other crest cells derived from the same axial level pass through the dorsolateral migratory pathway, being excluded from the region occupied by myotomes to form an arch-like pathway opening posteriorly (reviewed in [108]).

The above-described interface is formed primarily by the rostral-most somite and caudal-most part of the pharynx (Fig. 6), each representing, in the body of the pharyngula, the rostral end of the trunk environment and the caudal end of the head environment, respectively. Thus, the hypoglossal nerve, a trunk component, circumvents the pharyngeal arches by passing along the ventral curve of the interface, and the proximal part of the vagus nerve, a head component, passes along the dorsal curve to circumvent the trunk environment (Fig. 6). This curious morphological pattern reflects the vertebrate-specific plan of morphogenetic logics, showing unique sets of structures found only in vertebrates.

Around the abovementioned interface, vertebrate-specific structures appear, including the so-called neck (circumpharyngeal) muscles and the nerves to innervate these muscles [125, 144–146]. From the ventral part of the rostral somite-derived muscle plates, migrating muscle precursors are derived to migrate toward the ventral head region along the course of the hypoglossal nerve. These muscles, called the hypobranchial muscles, are vertebrate specific and are also found in cyclostomes, in a primitive form [147]. As the dorsal element of the circumpharyngeal muscles, the cucullaris muscle and its nerve, the accessory nerve, are recognized as derived traits that define gnathostomes. (See [148]; also see [128].) All these features arise in the unique embryonic environment established at the interface between the vertebrate head and trunk; this environment is not found in amphioxus (Fig. 6e).

Unlike the vertebrate pharyngeal arches, the amphioxus pharyngeal wall is independently separated medially from the wall that forms the ventral surface of the body. Myotomes penetrate into this pseudo-body wall, and there is no lateral plate-like continuous sheet of mesoderm. In this pattern of structural integration, unlike in vertebrates, the rostral “somites” never contact the pharyngeal wall, and therefore the head–trunk interface does not form. Thus, the latter interface is truly vertebrate specific, together with the structures patterned by this interface, including the hypobranchial and cucullaris muscles and the accessory, vagus, and hypoglossal nerves.

#### Somites and myotomes

There has been a long-standing prediction that the vertebrate head mesoderm evolved from the rostral segmented mesoderm of ancestral forms such as amphioxus ([129, 130, 149]. This idea originated from the so-called head segmentation theory that began as the vertebral theory of the skull proposed in the early nineteenth century by Oken [150] and Goethe [151], and before that by Vicq d’Azyr [152]. This originally transcendental idea was further strengthened by the discovery of epithelial coelom-like structures called head cavities in some primitive jawed vertebrates such as elasmobranchs and holocephalans ([153, 154]; reviewed in [155]). However, it has also been suggested that the head cavities represent a gnathostome synapomorphy, and that their epithelial segment-like configuration has nothing to do with the hypothetical head somites: there is no substantial difference in gene expression profiles between the head cavities and non-segmented, mesenchymal head mesoderm [156, 157] (reviewed in [155]).

On the basis of accumulated data on the gene expression profiles in developing paraxial mesoderm, however, it has become clear that amphioxus somites are not necessarily more similar to vertebrate somites than to head mesoderm, but they share gene expression profiles known to be specific to the vertebrate head mesoderm (reviewed in [156, 157]). Experimentally, as well, amphioxus somites are not necessarily closer to vertebrate trunk somites than to head mesoderm; it is possible that they represent an intermediate structure [158, 159]. (Also see [125] for a comparative embryological discussion).

The above arguments are based on the assumption that the common ancestor of amphioxus and vertebrates possessed an anteroposteriorly elongated body, with segmental mesodermal blocks throughout the entire axis. This assumption, however, has not been substantiated, although it may be relevant to the origin and homology of segments across bilaterians.

Masterman [160] once tried to identify the origin of metameric segments in three pairs of coelomic cavities derived from gut septations in the jellyfish. (Also see [161–163].) According to this scheme, the prototypic bilaterian mesodermal cavities have three components, the rostral-most one of which is found in the procoels in various bilaterian larvae, including the actinotrochs of phoronids, tornarian larvae of hemichordates, and auricularian larvae of echinoderms. Vertebrate embryos also fall into this category: The premandibular cavity is often assumed to be homologous to the procoel; the head mesoderm is homologous to the mesocoel and the entire somites to the metacoel [163]. If the early embryonic pattern of amphioxus is comparable to this scheme—especially the pattern of echinoderm larvae—then the anterior head diverticulum could represent the procoel of auricularians, and the rostral-most triangular somite would represent the mesocoel. (For other interpretations, see [164].) This level of comparison, however, potentially refers to the pan-bilaterian coelomic developmental program, not necessarily the chordate-specific morphotype. In addition, even if mesodermal homology between vertebrates and amphioxus could be established definitively, it would not mean that their body plans are identical, because the anteroposteriorly polarized distribution of the different generative constraints that yield the somitomeric and branchiomeric patterns in vertebrates is not present in amphioxus.

From the above, it is clear that the vertebrate body plan cannot have been derived from an amphioxus-like ancestor by continuous modification. The two animal groups possess conspicuous morphotypes that are distinctly different from each other: The homology of the mouth is lost, and the topographic relationship between somites and pharynx changes, during the early evolution of chordates, giving rise to different body plans. Chordates share only the notochord, postanal tail, and dorsal nerve chord as synapomorphies, because of the shared evolutionary history of dorsoventral inversion. However, the body plans of the three chordate lineages are as different as those found in each of the phyla among ambulacrarians, lophotrochozoans, or ecdysozoans. Given their distinct set of morphological patterns and elements, vertebrates are more appropriately classified as an independent phylum.

## Conclusion

Metazoan taxonomy and systematics, which are basic and important issues in zoology, provide basic information to help researchers interpret the grouping of various animal species. We believe that taxonomic interpretation must always be reexamined when new data are presented—especially new molecular data from different disciplines—as such comprehensive analyses are likely to inform more balanced decisions on classification. Here, we have discussed the possibility that Vertebrata should be recognized as an animal phylum. In light of the present subphylum rank of the Vertebrata, recognition of this possibility would facilitate future studies of the origin and evolution of vertebrates.
